# Changes in Plasma Carotenoid Concentrations during the AntioxObesity Weight Reduction Program among Adults with Excessive Body Weight

**DOI:** 10.3390/nu15234890

**Published:** 2023-11-23

**Authors:** Jadwiga Hamulka, Agnieszka Sulich, Magdalena Górnicka, Marta Jeruszka-Bielak

**Affiliations:** Department of Human Nutrition, Institute of Human Nutrition Sciences, Warsaw University of Life Sciences (SGGW-WULS), 02-787 Warsaw, Poland; agnieszka.sulich@gmail.com (A.S.); magdalena_gornicka@sggw.edu.pl (M.G.); marta_jeruszka-bielak@sggw.edu.pl (M.J.-B.)

**Keywords:** adults, carotenoids, intake, plasma, lipid profile, excessive body weight, weight reduction program

## Abstract

Plasma carotenoid concentrations are associated with antioxidant defense which might be disturbed in people with excessive body weight (EBW). This study aimed at evaluating the effect of a 6-week weight reduction program on plasma concentration of β-carotene, lycopene, and lutein/zeaxanthin in adults with EBW. A total of 130 adults were recruited for the study; 75 completed the program. Data on food consumption were collected with a 3-day recording method and a semi-quantitative FFQ. Body height, body weight (BW), waist circumference (WC), fat mass (FM), fat-free mass (FFM), abdominal subcutaneous adipose tissue (SAT), and visceral adipose tissue (VAT) were measured. Lipid profile, β-carotene, lycopene, and lutein/zeaxanthin were analyzed in blood. The AntioxObesity program resulted in a significant reduction in BW, WC, FM, SAT, and VAT. The mean plasma concentrations of β-carotene, lycopene, and lutein/zeaxanthin increased significantly after intervention. A reduction in FM above 4 kg significantly increased the concentration of β-carotene, lutein/zeaxanthin, and total carotenoids. An increase in carotenoid levels correlated with FM reduction, as fruit and vegetable intake remained unchanged. However, this effect may vary due to gender, HDL-cholesterol, body fat content, and obesity status in the weight loss process.

## 1. Introduction

Excessive body weight (EBW), including overweight and obesity, is one of the main factors affecting human health and plays an important role in the global burden of disease. Obesity is strongly associated with the development of cardiovascular diseases, type 2 diabetes, and several types of cancer, shortening human life expectancy and increasing the overall burden of disease worldwide [1].

It is estimated that reducing the BMI level of the population by 5% by 2030 will lower direct medical expenses related to obesity by EUR 495 million over the next 20 years [2]. Obesity and excessive amounts of adipose tissue are some of the main causes of increased oxidative stress, as well as intensification of inflammatory processes in an organism [3,4]. Moreover, recent studies have shown that oxidative stress also causes obesity through several mechanisms, such as stimulating the deposition of WAT (white adipose tissue), increasing the proliferation and differentiation of preadipocytes, and increasing the size of mature adipocytes. In turn, increased inflammation and oxidative stress lead to greater utilization of antioxidants, including carotenoids [5,6].

Carotenoids are fat-soluble pigments found in plants, fungi, bacteria, and algae, but their main sources are fresh and processed vegetables and fruits [7,8,9,10], which provide 80–90% of carotenoids in western diets. The matrix in which carotenoids are located, i.e., their physical distribution in various plant organelles (chloroplasts and chromoplasts) and complexes, is one of the main factors influencing their bioavailability. Carotenes are less accessible than xanthophylls, e.g., lutein and zeaxanthin due to their location and physical state in plant tissues [11,12]. Carotenoids can be obtained from food, but this is often insufficient due to the limited consumption of vegetables and fruits. Moreover, their total absorption largely depends on their absorption from the gastrointestinal tract, which may be inhibited due to the physicochemical properties of carotenoids. Approaches to solve these problems include developing staple foods enriched with carotenoids or seeking other sources like green algae [13,14]. Nutritional factors, such as fat and dietary fiber content, also influence carotenoids bioavailability. Fat consumed with meals increases the solubility of carotenoids by stimulating the production of bile salts and influencing the formation of larger micelles [11,15]. Carotenoids are absorbed only in their free form by the intestinal epithelium. In the small intestine, they are attached to lipid micelles, and then are transported in the blood by lipoproteins [16].

Although over several hundred different carotenoids are identified in food products, only β-carotene, lycopene, and lutein/zeaxanthin, α-carotene and β-cryptoxanthin are determined in blood and constitute 90% of all carotenoids in serum [17]. Carotenoids accumulate mainly in the liver and in the skin, adrenal glands, and reproductive tissues, and some of them, such as lutein and zeaxanthin, also accumulate in the eyes or brain [18,19,20,21]. They also have a high affinity for adipose tissue, and adipose tissue carotenoid content is related to both dietary intake and other tissue concentrations. Thus, carotenoid content in this tissue is considered a good long-term indicator of the dietary intake of those compounds [21,22,23]. So far, the mechanisms of specific absorption and accumulation of carotenoids in adipose tissue are not known, but recent results indicate that the total content of β-carotene in adipose tissue is similar (130 ± 70 µmol/person), regardless of BMI or its different intake level [24]. Moreover, some studies suggest that putative transporters or facilitators might be involved, as the carotenoid uptake by adipose tissue was independent of the carotenoid’s physical and chemical properties [25].

It is worth noting that carotenoids have a potentially beneficial effect on human health. They have a positive effect on many functions in the body, mainly throughout their antioxidant, anti-inflammatory, and immunomodulatory properties, as well as via the modulation of gene expression or regulation of intercellular communication [26,27,28,29,30,31]. A positive association between higher carotenoid concentrations and a lower risk of chronic disease has been illustrated by epidemiological studies, with β-carotene and lycopene being negatively associated with the risk of cardiovascular disease, lutein and zeaxanthin with eye diseases and cognitive function, or beta-cryptoxanthin with bone diseases [17,18,31,32]. Carotenoids are essential in regulating oxidative metabolism and reducing cellular differentiation in the treatment of obesity. As presented by Kurniawan et al. [13] in in vitro tests, carotenoids present in the extract of green algae (*Caulerpa racemose*) showed antioxidant activity, including inhibiting TNF-α and mTOR, and increasing AMPK levels, which may be a promising anti-obesity and anti-inflammatory agent.

Moreover, the relationship between the nuclear receptor superfamily and carotenoids may indicate carotenoid involvement in anti-obesity mechanisms of action [5,33]. Fat-soluble carotenoids have been shown to be present in lipid droplets in adipocytes and to influence lipid absorption and transport, indicating a correlation between carotenoids and EBW [33]. Ample evidence exists that carotenoids, as highly fat-soluble compounds, accumulate in fat-collecting tissues, as well as in adipose tissue itself. It is believed that the content of adipose tissue in an organism may significantly affect the distribution of these compounds to other tissues and may also affect their concentration in the blood [17,23,28,34].

In the available literature, very few studies assessed the impact of fat tissue reduction on the nutritional status of carotenoids and changes in their blood concentration. Therefore, the aim of this study was to determine the impact of a weight reduction program on blood carotenoid concentration in women and men with overweight and obesity.

## 2. Materials and Methods

### 2.1. Ethics Approval

This study was approved by the Bioethics Committee of the National Food and Nutrition Institute (resolution No. 1805/2011) and was conducted in compliance with the Declaration of Helsinki. All subjects gave their written informed consent to participate in the study. Data obtained during the intervention were confidential and restricted to the participating investigators.

### 2.2. Study Design and Participants

**Figure 1:**
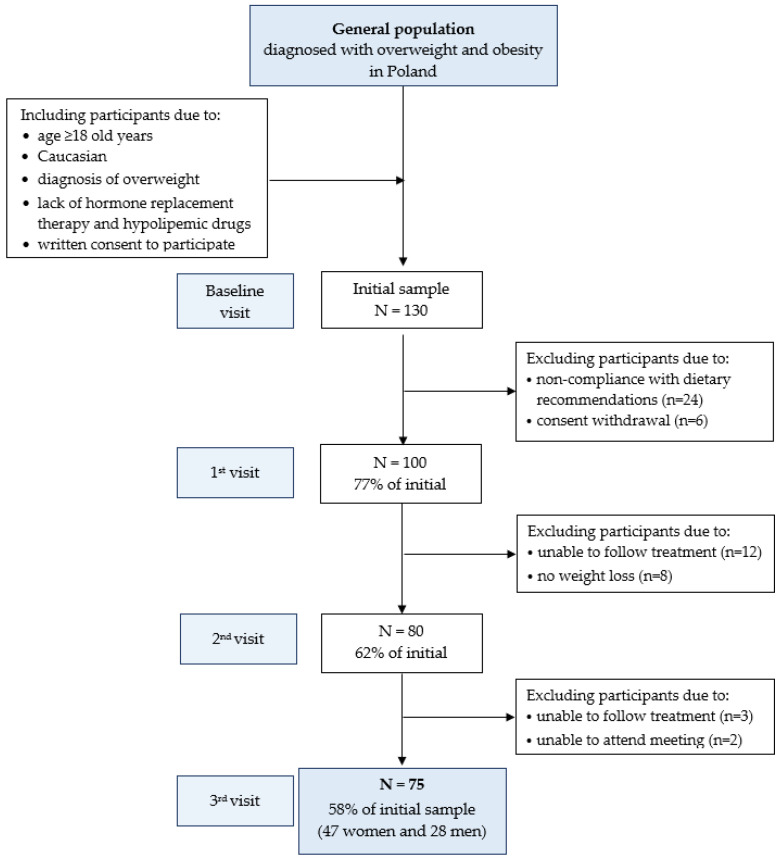
Flowchart: study design and data collection.

The present study is a part of a larger intervention study conducted in 2012–2018, concerning the impact of weight reduction on the antioxidant status in adults with excessive body weight—AntioxObesity program [35]. Briefly, in the study, 130 males and females who fulfilled the following inclusion criteria were involved: adults (≥18 years); overweight or obese according to WHO definition: BMI ≥ 25 kg/m^2^ and WC: men: >94 cm, women: >80 cm [36,37]; lack of hormone replacement therapy and hypolipemic drugs; consent to participate. Participants receiving pharmacological treatment or those diagnosed with chronic diseases, allergies, food intolerances, those with large fluctuations in body weight in the last 6 months, and people taking medications that may have affected the results of this study as well as those taking supplements with carotenoids were excluded from the study. In the case of women, the exclusion criteria were also pregnancy, lactation, and menopause. Although a total of 130 subjects were initially screened, only 75 completed the program, 47 women and 28 men (mean age 34.7 ± 9.0 years)—Figure 1.

#### 2.2.1. AntioxObesity Program

The AntioxObesity weight loss program involved the development, implementation, and evaluation of the efficacy of a therapeutic program for overweight and obese adults and included comprehensive education on nutrition and physical activity [35]. The main assumption of the AntioxObesity weight loss program was to investigate how the process of weight reduction influenced biochemical parameters (lipid profile) and the plasma concentration of lipid antioxidants—α-tocopherol and carotenoids (β-carotene, lycopene, lutein/zeaxanthin)—without changes in the dietary intake of these compounds. Therefore, in the recommendations, particular attention was paid to the appropriate selection of fruits and vegetables, so that the intake of antioxidants was maintained at a similar level throughout the whole program.

**Figure 2:**
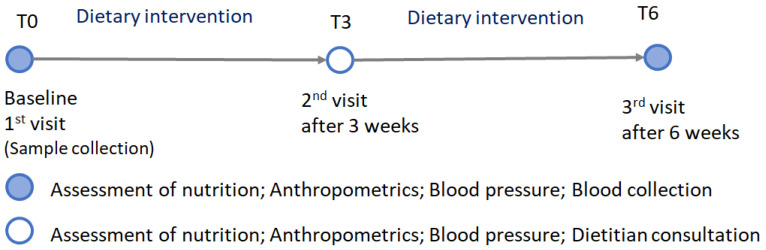
Timeline and activities of the AntioxObesity weight reduction program.

The aim of the AntioxObesity program was weight/fat loss through a reduced energy diet during a 6-week period. Participants visited the research center 3 times: at baseline (T0), at mid-term—after 3 weeks from the baseline (T3)—and at the end of the whole dietary intervention—after 6 weeks (T6) (Figure 2). During all three visits, dietary data were collected, anthropometric measurements were taken, and blood pressure was measured. Blood was collected two times—at T0 and T6.

#### 2.2.2. Compliance with Dietary Treatment

According to the main goals of dietary intervention, the dietary compliance criteria were: (1) the energy value of the diet reduced by approximately 500–700 kcal—20–25% in relation to the initial energy value of each person, individually determined according to the BMI value; (2) adequacy of carbohydrates intake (energy from carbohydrates between 50 and 55% ± 5%); (3) adequacy of protein intake (energy from protein between 15 and 20% ± 5%); (4) adequacy of fat intake (energy from fat between 25 and 30% ± 5%); (5) adequacy of meal frequency, based on 3 main meals (breakfast, lunch, and dinner) and 2 snacks (mid-morning and mid-afternoon). Participants, who achieved at least 3 out of those 5 dietary goals were considered as showing “global compliance”.

### 2.3. Data Collection and Procedures

#### 2.3.1. Dietary Assessment

Data on food consumption were collected with a 3-day recording method and a semi-quantitative food frequency questionnaire. A dietary record was conducted according to widely accepted and applied rules [38]. Respondents were trained how to self-report all foods and beverages consumed daily to provide reliable estimates of dietary intake. Assessment of the diets was based on the self-reported data in dietary records made in three typical, nonconsecutive random days (two weekdays and one weekend day). The diary was checked by a nutritionist in the participants’ presence. The nutritionist asked for detailed information about the foods and drinks recorded, such as preparation methods and portion sizes using food models of products and dishes [39]. When necessary, the food diary was corrected by the nutritionist during the visit. After the review, food intake data were converted to food volume/weight (in mL or g). These data were entered into a nutritional software program (Dieta ver. 5.0; National Food and Nutrition Institute, Warsaw, Poland) to evaluate daily energy and macronutrients. The intake of lycopene and lutein/zeaxanthin and β-carotene was assessed using the National Nutrient Database for Standard Reference of the United States Department of Agriculture [40]. The mean values from three recorded days were used for further analysis. Current dietary recommendations were used to assess the adequacy of energy and selected nutrients intake [41].

#### 2.3.2. Anthropometry Measurements

Body height, body weight, and waist and hip circumferences were measured using standardized procedures according to the International Society for Advancement of Kinanthropometry (ISAK) International Standards for Anthropometric Assessment guidelines [42,43]. Professional equipment and a measuring tape were used. Body weight (BW) was measured with the electronic digital scale to the nearest 0.1 kg (SECA 799, Hamburg, Germany). Height (H) was measured using a stadiometer with the head in the horizontal Frankfurt plane and recorded with a precision of 0.1 cm (SECA 220, Hamburg, Germany). Waist circumference (WC) was measured with a stretch-resistant tape that provides constant 100 g tension (SECA 201, Hamburg, Germany) at the midway point between the iliac crest and the costal margin (lower rib) on the anterior axillary line in a resting expiratory position. Hip circumference (HC) was measured around the widest part of the buttocks, with the tape parallel to the floor. Body mass index (BMI) was calculated as weight (kg)/height (m^2^). BMI was categorized according to WHO, taking values above 25 kg/m^2^ as EBM [44].

Bioelectrical impedance analysis (BIA) (Maltron BioScan 920 ver.1.1) was used to assess fat mass (FM) and fat-free mass (FFM), including subcutaneous adipose tissue (SAT) and visceral adipose tissue (VAT). BIA was performed under standardized conditions according to the manufacturer’s protocol. All measurements were taken with light clothing and with metal objects (e.g., jewelry, keys) removed. Whole body BIA measurements were performed by placing two adhesive single-use skin electrodes (purchased from Maltron International Ltd., Rayleigh, UK) on the right hand and foot, respectively, on the patient when lying in supine position. The device applies a current of 400 mA at a constant frequency of 50 kHz. The VAT and SAT measurements were performed in the standing position with four pairs of electrodes positioned on the trunk. Measurements were taken at a frequency of 50 kHz, with an impedance range of 5–1100 Ω.

#### 2.3.3. Blood Samples and Biochemical Analyses

Fasting venous blood samples were collected after an overnight fast (12 h) in the morning (9–10 a.m.) using standard techniques. The procedure of blood sample collection and plasma obtaining were described previously [35]. In brief, 10 mL of blood samples were taken with minimal stasis and maintained at 4 °C until plasma was separated for biochemical analyses. Plasma samples were collected after centrifugation (1000× *g* for 10 min at 4 °C) and stored frozen (at −80 °C) until further analysis conducted within 2 months.

All biochemical analyses were determined with standard methods by a certified laboratory. Lipid profile (total cholesterol, HDL-cholesterol, and triglycerides) was determined with standard enzymatic analyses using commercial HYDREX kits (product numbers: total cholesterol—HXB104; HDL-cholesterol—HXB106; triglycerides—17628). LDL level was calculated using the Friedwald formula [45]. The results were expressed as mM/L.

Plasma carotenoids concentration was assessed with high-performance liquid chromatography (HPLC) (Gilson Company, Middleton, WI, USA), with UV-VIS detector, after extracting carotenoids with organic solvents. Carotenoids concentrations were analyzed in plasma with the adopted methodology of Wu et al. [46]. Firstly, 0.5 mL of 99.9% ethanol containing 0.1% butylhydroxytoluene (BHT) as an antioxidant agent was added to 0.5 mL of blood plasma. Each sample was mixed with vortex for 30 s and set aside for 15 min. Then, 1 mL of hexane containing 0.02% BHT was added and further mixed for 2 min to extract carotenoids. The extracts were applied to a C18 RP chromatographic column (4.6 × 250 mm; 5 µm) from Vydac, with a pre-column from the same company (Vydac 201TP54 Company, Hesperia, CA, USA). Carotenoids were analyzed at a wavelength of 470 nm. Acetonitrile/hexane/dichloromethane/methanol mixed in the proportions 50:20:20:10 was used as the developing mixture. The flow rate of the developing mixture was 1.0 mL/min. Carotenoids concentrations were related to standard curves prepared with Sigma Aldrich standards, expressed in nM/L, and compared with the estimated values (Table 1).

All data from T0 and T6 were used to determine the changes in plasma carotenoids due to the 6-week AntioxObesity weight loss program and their influence on carotenoids status in relation to BMI, FM, VAT, SAT, and lipid profile in adults with EBW.

### 2.4. Statistical Analysis

Qualitative data were presented as the percentage of people (%) in each category, and the Chi-square test was applied to detect the statistical differences between groups. The parameters analyzed during the weight reduction program and the data on dietary intake were presented as mean and standard deviation. The distribution of quantitative data was checked with the Shapiro–Wilk W test. As the variables were not normally distributed (also after using the logarithm of the data), non-parametric tests were used for comparisons between/among groups: the Mann–Whitney *U* test (for 2 groups of independent variables) or the Kruskal–Wallis test (for more than two groups of independent variables). To verify differences between mean values, post-hoc analysis with Tukey’s test was used. For the changes in parameters during the AntioxObesity weight reduction program, non-parametric tests were used for dependent variables: Wilcoxon signed-rank test (for two variables) and Friedman’s rank test (for three variables). Spearman’s rank correlation coefficient was used to assess the correlations between variables.

Statistical analysis was conducted using Statistica ver. 13.3 PL (TIBCO Software Tulsa, OK, USA; StatSoft. Krakow, Poland). A *p*-value < 0.05 was considered to indicate statistical significance.

## 3. Results

The study group consisted of 75 people, including 47 women and 28 men with an average age of 34 ± 9 years, from Warsaw (75%) and the surrounding areas (25%). The characteristics of the study population are presented in Table 2. The majority of participants had higher education (72%) and were professionally active (81%). Smoking was declared by 12% of people, and eight cigarettes were smoked daily, on average. A low or moderate level of physical activity was declared by 93% of subjects. Neither sociodemographic nor lifestyle characteristics differed significantly between women and men.

The mean body weight ranged from 67 to 141 kg and was significantly higher in men than women by 21%, on average. The initial mean BMI value was 32 ± 5 kg/m^2^ and did not differ between both genders. Most people (63%) had obesity (BMI ≥ 30), while the remaining 37% were overweight (Table 2).

The mean dietary energy intake at T0 was 2006 kcal per day, and decreased by 400 and 500 kcal, in T3 and T6, respectively (Table 3). Similarly, the total fat intake was significantly reduced by 30% in T3 and by 37% in T6. Both energy and fat intake were significantly higher in men than women at all program stages. The mean energy percentage from fat was within the reference values (20–35%) for both subgroups and at all measurement points. Although it did not differ significantly between men and women or among three program stages, a decrease by 3% at T3 and by 4% at T6 in comparison with T0 was observed.

The mean intakes of β-carotene, lycopene, and lutein/zeaxanthin were similar at each stage of the weight loss program (Table 3). Moreover, gender, BMI, or the scale of weight loss did not significantly affect the intake of those compounds.

The AntioxObesity program resulted in a significant reduction in anthropometric parameters, like body weight, BMI, and waist circumference, (Table 4). Body weight decreased by 3.9 kg after 6 weeks, on average, and the weekly weight loss was approximately 0.6 kg. According to BMI, the percentage of people with obesity decreased from 63% at T0 to 47% at T6; moreover 9% of people achieved normal body weight at T6.

The mean WC at T0 was 91 cm in women and 101 cm in men (Table 4). In 83% of women and 75% of men, WC values indicated the presence of abdominal obesity. After 6 weeks of the AntioxObesity program, WC decreased by approximately 5 cm on average, regardless of gender.

Body composition also had improved after the AntioxObesity program. A significant decrease in total FM was found (by 3.3 kg, on average), with unchanged mean values of FFM, both in women and men (Table 4). The FM declined by 8% in women and 10% in men. A fat tissue reduction of 4 kg or more was found in approximately 54% of men and 47% of women. A higher percentage in VAT than in SAT loss was observed in the general population and in both genders (Table 4). The reduction in SAT was significant in the general population and in men, while the changes in women were not significant and amounted to 4%.

The greatest reduction in the values of anthropometric parameters occurred during the first 3 weeks of the program.

In Table 5, changes in lipid profile during the AntioxObesity program are presented. Mean values of total cholesterol and LDL-cholesterol exceeded the reference value in the general population at T0 by 4% and 7%, respectively. At T6, both parameters decreased significantly by 7% and 8%, on average, mainly due to the changes in women. On the contrary, neither HDL-cholesterol nor triglycerides changed during the program.

Mean plasma β-carotene concentration increased significantly from 612 nM/L at T0 to 651 nM/L at T6 in the total group (Table 6), and such changes were recorded in 68% of the subjects. A higher (and significant) raise in plasma β-carotene level was observed in women (72 nM/L) than in men (37 nM/L). Mean plasma concentrations of β-carotene increased during the AntioxObesity program, regardless the BMI or FM categories, although the differences for FM reduction below 4 kg did not reach statistical significance.

The mean plasma lycopene levels have not changed between T0 and T6 (Table 6). Factors such as gender, BMI, or FM reduction categories did not significantly affect the plasma concentration of lycopene in the subjects.

During the AntioxObesity program, plasma lutein/zeaxanthin concentrations raised in 65% of the subjects. The mean increase between T0 and T6 equaled 14%, 13%. and 16% in total population, women, and men, respectively; although, in men, it was not significant (Table 6). Considering the BMI and the FM reduction categories, a significant increase in plasma lutein level was noted—on average by 17% in respondents with overweight and by 16% in those with higher FM reduction.

Analyzing the relationship between plasma carotenoids changes and FM reduction, we found that FM reduction above 8% was associated with a significant increase in β-carotene and lutein/zeaxanthin concentrations, but not lycopene (Table 7). Significant increases in total carotenoids concentrations were also demonstrated, regardless of the degree of FM reduction.

Only lutein inversely correlated with BMI (r = −0.262) and FM (r = −0.257) in the entire group after completing the weight loss program. A positive correlation was found between lycopene and VAT (r = 0.344) as well as between lycopene and SAT before (r = 0.335) and after (r = 0.329) the program, but only in women. Significant positive correlations between lycopene and total cholesterol in the whole population (r = 0.231), and lutein and total cholesterol (r = 0.306) in the entire group as well as in women (r = 0.294) were found. HDL-cholesterol correlated positively with lycopene (r = 0.236) and with lutein (r = 0.311) in the entire group as well as in women (r = 0.353 and r = 0.355, respectively) after the program. A positive correlation was found between lycopene and triglycerides before the program in the entire group (r = 0.240) as well as in women (r = 0.309), and after the intervention, but only in women (r = 0.315).

## 4. Discussion

With the implementation of a body weight reduction program (the AntioxObesity program) with a low-energy diet and maintained carotenoids intake (like the amounts before the program), significant decreases in anthropometric parameters such as body weight, BMI, waist circumference, and fat tissue content, including subcutaneous and visceral adipose tissue, were observed. A significant raise in plasma carotenoids concentration and a decrease in blood total cholesterol and LDL-cholesterol were also demonstrated. Plasma β-carotene and lutein increased on average by 9% and 14%, respectively. Moreover, the greatest reduction in FM (above 8%) was associated with a significant increase in plasma β-carotene and lutein levels. In people with body weight lost ≥4 kg, a higher percentage of FM reduction was associated with a higher increase in plasma β-carotene concentration. However, no such trends were observed for lycopene.

In our study, the intake of β-carotene, lycopene, and lutein was comparable throughout the entire period of the weight loss program and equaled approx. 5, 4, and 2 mg per day, respectively, for the total population. Similar intakes of those carotenoids were also observed in other studies conducted among Polish adult populations, ranging from 4 to 6 mg/day for β-carotene, 2–7 mg/day for lycopene, and 2–3 mg/day for lutein [47,48,49,50].

In a review of 142 studies involving adults, the mean intake of β-carotene, lycopene, and lutein was comparable to the levels evaluated in our own study and equaled 4, 5, and 2 mg per day, respectively [51]. Considering estimated normal daily carotenoid intakes [31], we can conclude that our participants at each stage of the program had an adequate intake of β-carotene and lycopene, but too low an intake of lutein (2.1 vs. 4.6 mg/day). Despite adequate consumption, the plasma level of lycopene did not reach the desired values [31].

Plasma carotenoids are biomarkers of both vegetable/fruit and carotenoids intake [52]. However, it is worth noting that the bioavailability and absorption of carotenoids are limited by factors such as their dietary amount and sources, food matrix and carotenoid location, food heating and processing, season, and food composition, specifically the intake of fat, dietary fiber, protein, and other compounds [12,53,54]. The existing literature confirms the impact of external or host-related factors like obesity, including BMI and other anthropometric parameters, on the blood carotenoids concentration, regardless of other factors, such as fruit and vegetables consumption, fat and dietary fiber intake, alcohol and dietary supplements usage, smoking, blood lipid parameters, gender, and microbiome, as well as genetic differences, including single nucleotide polymorphisms regulating carotenoid metabolism [12,48,55,56,57,58,59,60]. After digestion and absorption, carotenoids are transported in the bloodstream bound to lipoproteins, where carotenes dominate over the LDL fraction and xanthophylls are almost evenly distributed between LDL and HDL. Consequently, changes in the lipoprotein pattern, due to external or host-related factors, may modulate tissue distribution of carotenoids [61,62]. Moreover, as research results indicate, higher plasma carotenoid concentrations were also associated with a favorable lipid profile in elderly people with excessive body mass and metabolic syndrome [63]. It was explained with an increase in the hydrolysis of long-chain fatty acids and the induction by carotenoids of some enzymes associated with mitochondrial and peroxisomal β-oxidation. On the other hand, as Amengual et al. [64] pointed out, carotenoids are cleaved by two carotenoid oxygenases, BCO1 and BCO2, with BCO1 being the only enzyme capable of producing vitamin A in mammals, and the activity of BCO1, not the concentration of β-carotene itself, acts as a modulator of non-HDL cholesterol, affecting total cholesterol levels. Moreover, individual carriers of the major T allele variant oBCO1-rs6564851 show lower total and non-HDL cholesterol concentrations, regardless of age, sex, BMI, retinol intake, and total carotenoid or β-carotene intake.

At the stage before dietary intervention, we found significant positive correlations between lycopene and triglycerides in the entire group and in women; lycopene and HDL-cholesterol in women; and lutein and HDL-cholesterol in the entire group, regardless of gender. After completing the program, positive correlations were noted between lycopene and lutein, and total cholesterol and HDL-cholesterol in the general population and in women. A positive correlation between total blood carotenoids and the HDL-cholesterol was also demonstrated by other authors [55,65,66,67]. As mentioned above, carotenoids are transported in various proportions by lipoproteins, with β-carotene and lycopene occurring mostly in LDL (58–73%) and HDL (17–26%), and lutein and zeaxanthin in HDL (53%) and LDL (31%) [68]. In our study, only plasma lycopene and lutein concentrations were positively correlated with circulating HDL-cholesterol, and this relationship was confirmed only in women. Gender differences in lipid and lipoprotein metabolism are well known, with women usually having lower LDL-cholesterol and higher HDL-cholesterol than men, although it was not the case in our group. In the present study, plasma LDL-cholesterol concentrations were similar in men and women, but LDL concentration decreased significantly only in women, because of weight loss. This presumably may explain the observed correlations. A possible interpretation for this observation may be related to the differences in the physical properties of lipoprotein particles; men also have smaller and denser LDL and HDL particles compared to women, which are characterized by a reduced content of carotenoids [68]. Moreover, in the study conducted among 108 overweight people aged 40.7 ± 12.5 years, women, when compared to men, had higher blood concentrations of all analyzed carotenoids: α-carotene, β-carotene, β-cryptoxanthin, lycopene, and lutein together with zeaxanthin by 36%, 46%, 18%, 5%, and 8%, respectively [67]. This confirms previous observations that women usually have higher blood concentration of carotenoids than men. This may result from several causes: differences in fruit and vegetable intake, differences in the efficiency of carotenoid absorption, differences in total blood volume (which is smaller in women, leading to a higher blood concentration of carotenoids after consuming similar amounts of carotenoids), and finally, differences in metabolism [62].

Our results showed that people with obesity (BMI ≥ 30 kg/m^2^) had lower blood carotenoid levels than subjects with overweight. However, significant differences were determined only for lutein/zeaxanthin, both before and after the weight loss program. A higher BMI was associated with lower amounts of circulating carotenoids, although not all studies included differences in carotenoids intake [12,69]. These relationships may occur due to greater fat mass which in turn is associated with higher oxidative stress, which finally reduces circulating carotenoid concentrations, or with abdominal adipose tissue, where carotenoid accumulation is the greatest [12,22,70].

Those observations may be confirmed by the tendency of a decrease in plasma carotenoids concentration with the increase in adipose tissue percentage recorded in our study. The content of adipose tissue was negatively correlated with the plasma lutein level after the completion of the body weight reduction program in women. However, a positive correlation was found between lycopene and VAT after completing the weight loss program as well as between lycopene and SAT before and after the program, also only in women. Similar results were presented by Matsumoto et al. [71], indicating that higher levels of lutein and β-carotene in serum were only associated with lower levels of visceral fat area in women, while the observed positive correlation between lycopene and visceral fat (in men) could be explained by the low level of dietary intake and the influence of other nutrients in dishes. Plasma levels of most carotenoids were inversely correlated with fat mass and general and central obesity [61,72], and with weight loss, serum lutein, and zeaxanthin concentrations increase [23]. This is also corroborated by our results, where the increase in plasma lutein concentration during the weight loss program was higher than that of β-carotene and lycopene.

Since obesity is associated with chronic inflammation, an association with greater carotenoid degradation cannot be ruled out either, but this remains hypothetical. On the other hand, it is possible that the body adapts to increased oxidative stress by increasing circulating antioxidants levels in the plasma [61]. Adipose tissue is a good long-term indicator of the dietary intake of carotenoids and an important place for the accumulation of carotenoids, where lycopene and β-carotene dominate (more than 1/2 and approx. 1/3 of total carotenoids, respectively) [23]. Although carotenoid content per gram is higher in other organs, adipose tissue contains the greatest amount and is considered to be used for the storage of carotenoids. Similarly, subcutaneous adipose tissue is a storehouse of carotenoids and a part of a balanced carotenoid distribution system in adipose tissue stores; therefore, their reduction may be related to changes in plasma concentration of carotenoids. It is hypothesized that a greater amount of adipose tissue with a high affinity for storing carotenoids reduces the release of carotenoids into the bloodstream or increases their uptake from the circulatory system, probably through LDL receptors [61]. Unfortunately, knowledge about the role of participants in the regulation of carotenoid uptake/release in this tissue is poor [61].

To summarize, it should be stated that although diet is the main factor influencing the blood concentration of carotenoids, excessive body weight, including the content of adipose tissue and its distribution in the body (abdominal fat), is associated with their reduced level in the blood, specifically in people with obesity. This relationship may result from the increased accumulation of these compounds in adipose tissue. Additionally, adipose tissue may influence the distribution of carotenoids in other tissues, affecting their availability to the organism.

Results obtained in our study and by other authors suggest that the reduction in adipose tissue increases the blood concentration of selected carotenoids, specifically β-carotene and lutein/zeaxanthin. In light of current knowledge, higher carotenoid concentrations in blood are associated with better antioxidant protection of an organism and reduced morbidity due to increased oxidative stress. However, this requires further research in this area to learn the mechanisms of the distribution of these compounds in the human body and the influence of other factors on this process.

This study shows that gender, HDL-cholesterol, body fat content, and obesity status may differentiate the plasma concentration of carotenoids in the process of body weight reduction. Our results suggest that using crude circulating carotenoid concentrations without taking these factors into account leads to erroneous inferences. Better understanding the factors influencing plasma carotenoid concentrations will allow for the application of them more accurately as nutritional biomarkers to assess dietary intake. In addition, a strength of the study is the comprehensive analysis of body composition, including the measurement of SAT and VAT with BIA. In many previous studies, the established relationship between carotenoids and obesity was limited only to BMI- or waist circumference-based assessments. As Matsumoto et al. [71] confirmed, for a central obesity assessment, measuring the visceral fat area is more reliable than measuring waist circumference. Chung et al. [22] also pointed out that the total concentration of carotenoids in the abdominal cavity is higher than in the buttocks or thighs, and adipose tissue may influence the distribution of carotenoids to other tissues. Moreover, another strength of this study is the management of dietary data collection by professionals and the checking of data with face-to-face interviews during the whole study. To improve the accuracy of participants’ estimation of food weight, we provided food photos of standard food portion sizes to facilitate the assessment of food weight.

On the other hand, there were a few limitations to this study. Firstly, food composition databases rarely show data on individual carotenoids, and the important issue of bioavailability is also missing [73]. This appears to be key information because different carotenoids and forms of carotenoids exhibit different bioavailability (i.e., free vs. ester forms and xanthophylls vs. carotenes) [74,75]. Accurate measurement of intake is challenging, and current dietary assessment methods have strengths and limitations in estimating carotenoid intake. Further research is needed to improve the assessment of dietary intake and establish biologically relevant dose–response relationships in the context of individual variability to advance our understanding of diet, disease risk, and health promotion, specifically in individuals with excessive body weight.

## 5. Conclusions

It was observed that the increase in plasma carotenoid levels correlated with a reduction in fat mass, as fruit and vegetable intake remained unchanged. However, this effect may vary due to gender, HDL-cholesterol, body fat content, and obesity status in the weight loss process. More precise knowledge in this matter will help to develop personalized dietary guidelines. Nevertheless, this requires further research to understand the mechanisms and determinants of the distribution of these compounds in an organism.

## Figures and Tables

**Table 1:** Estimated values for carotenoids in plasma and diet [31].

Carotenoids	Total	β-Carotene	Lycopene	Lutein and Zeaxanthin
plasma (nM/L)	1725	500	600	330
diet (mg/d)	11.8	4.1	2.2	4.6

**Table 2:** Characteristics of the study group.

Variables	Total Group n = 75	Women n = 47	Men n = 28	*p*-Value
Age (years)	34.7 ± 9.01	33.9 ± 9.1	36.0 ± 8.9	NS
Place of living (%)				NS
>100,000 residents	74.7	70.2	82.1	
<100,000 residents	25.3	29.8	17.9	
Education (%)				NS
secondary	28.0	29.8	25.0	
university	72.0	70.2	75.0	
Professionally active (%)				NS
yes	81.3	83.0	78.6	
no	18.7	17.0	21.4	
Smoking (%):				NS
yes	12.0	10.6	14.3	
no	88.0	89.4	85.7	
Physical activity (%)				NS
high	6.7	2.0	14.3	
moderate	33.3	36.2	28.6	
low	60.0	61.8	57.1	
High (m)	1.7 ± 0.1	1.7 ± 0.1	1.8 ± 0.1	<0.001
BW (kg)	93.3 ± 17.2	86.5 ± 14.5	104.7 ± 15.2	<0.001
BMI (kg/m^2^)	32.0 ± 4.7	31.7 ± 4.8	32.5 ± 4.5	NS
BMI categories (%)				NS
25–29.9 kg/m^2^	37.3	40.4	32.1	
≥30 kg/m^2^	62.7	59.6	67.9	

BW, body weight; BMI, body mass index; NS, not significant.

**Table 3:** Changes in energy, fat, and carotenoids intake during the AntioxObesity program.

Variables	Group	Stage of the AntioxObesity			*p*-Value *
		T0	T3	T6	
Energy value (kcal/d)	Total	2006 ± 605 ^a^	1591 ± 454 ^b^	1499 ± 444 ^c^	<0.001
	Women	1811 ± 537 ^a^	1445 ± 406 ^b^	1345 ± 366 ^c^	<0.001
	Men	2335 ± 576 ^a^	1836 ± 431 ^b^	1757 ± 449 ^b^	0.001
	*p*-value **	<0.001	<0.001	<0.001	
Fat (g/d)	Total	73.1 ± 32.7 ^a^	51.5 ± 25.7 ^b^	46.0 ± 19.3 ^b^	<0.001
	Women	63.6 ± 28.9 ^a^	46.3 ± 24.8 ^b^	41.1 ± 16.2 ^b^	<0.001
	Men	88.9 ± 33.2 ^a^	60.2 ± 25.4 ^b^	54.3 ± 21.3 ^b^	<0.001
	*p*-value **	<0.001	0.02	0.006	
Fat (% energy)	Total	31.1	28.0	26.9	NS
	Women	29.7	27.4	27.0	NS
	Men	33.5	28.9	26.9	NS
	*p*-value **	NS	NS	NS	
β-carotene (mg/d)	Total	4.5 ± 2.5	4.8 ± 2.5	4.7 ± 2.3	NS
	Women	4.6 ± 2.6	4.8 ± 2.4	4.8 ± 2.3	NS
	Men	4.4 ± 2.3	4.8 ± 2.5	4.6 ± 2.5	NS
	*p*-value **	NS	NS	NS	
Lycopene (mg/d)	Total	3.6 ± 2.2	3.8 ± 2.3	3.8 ± 2.3	NS
	Women	3.4 ± 2.0	3.7 ± 2.2	3.7 ± 2.4	NS
	Men	3.8 ± 2.5	4.0 ± 2.5	3.9 ± 2.5	NS
	*p*-value **	NS	NS	NS	
Lutein/ zeaxanthin (mg/d)	Total	2.1 ± 1.3	2.1 ± 1.2	2.2 ± 1.2	NS
	Women	2.2 ± 1.3	2.3 ± 1.3	2.3 ± 1.3	NS
	Men	2.0 ± 1.1	1.9 ± 0.9	2.0 ± 1.0	NS
	*p*-value **	NS	NS	NS	

*, Friedman’s rank test; **, Mann–Whitney *U* test; NS, not significant; different letters indicate that the samples are significantly different at *p* < 0.05.

**Table 4:** Changes in body size and composition during the AntioxObesity program.

Variables	Group	Stage of the AntioxObesity			*p*-Value *	Changes %
		T0	T3	T6		
BW (kg)	Total	93.3 ± 17.2 ^a^	90.5 ± 17.1 ^b^	89.4 ± 16.9 ^c^	<0.001	↓ 4.2 ± 3.0
	Women	86.5 ± 14.5 ^a^	84.0 ± 14.5 ^b^	82.9 ± 14.3 ^c^	<0.001	↓ 4.2 ± 2.6
	Men	104.7 ± 15.2 ^a^	101.4 ± 15.6 ^b^	100.3 ± 15.5 ^c^	<0.001	↓ 4.3 ± 3.5
	*p*-value **	<0.001	<0.001	<0.001		
BMI (kg/m^2^)	Total	32.0 ± 4.7 ^a^	31.1 ± 4.6 ^b^	30.7 ± 4.5 ^c^	<0.001	↓ 4.2 ± 3.1
	Women	31.7 ± 4.8 ^a^	30.8 ± 4.7 ^b^	30.4 ± 4.6 ^b^	<0.001	↓ 4.2 ± 2.8
	Men	32.5 ± 4.5 ^a^	31.4 ± 4.4 ^b^	31.1 ± 4.4 ^b^	<0.001	↓ 4.3 ± 3.6
	*p*-value **	NS	NS	NS		
WC (cm)	Total	94.8 ± 12.3 ^a^	91.8 ± 11.9 ^b^	89.9 ± 11.9 ^c^	<0.001	↓ 5.1 ± 4.0
	Women	90.0 ± 10.5 ^a^	87.1 ± 9.8 ^b^	85.1 ± 9.7 ^c^	<0.001	↓ 5.3 ± 4.2
	Men	102.8 ± 11.0 ^a^	99.6 ± 11.1 ^b^	97.9 ± 11.1 ^c^	<0.001	↓ 4.8 ± 3.7
	*p*-value **	<0.001	<0.001	<0.001		
FFM (kg)	Total	53.6 ± 11.6	53.8 ± 12.3	53.1 ± 11.3	NS	↓ 0.7 ± 3.3
	Women	45.4 ± 3.6	45.5 ± 3.3	45.2 ± 3.4	NS	↓ 0.4 ± 3.6
	Men	67.4 ± 5.6	67.8 ± 8.6	66.5 ± 6.0	NS	↓ 1.4 ± 2.6
	*p*-value **	<0.001	<0.001	<0.001		
FM (kg)	Total	39.6 ± 13.3 ^a^	36.6 ± 13.0 ^b^	36.3 ± 13.3 ^b^	<0.001	↓ 8.9 ± 7.8
	Women	41.0 ± 14.0 ^a^	38.4 ± 13.9 ^b^	37.8 ± 14.0 ^c^	<0.001	↓ 8.3 ± 7.4
	Men	37.3 ± 12.1 ^a^	33.5 ± 10.8 ^b^	33.7 ± 11.9 ^b^	<0.001	↓ 9.9 ± 8.3
	*p*-value **	NS	NS	NS		
SAT (cm^2^)	Total	248.0 ± 82.5 ^a^	240.1 ± 82.3 ^a^	228.2 ± 86.6 ^b^	0.002	↓ 7.2 ± 21.7
	Women	242.0 ± 75.1	236.7 ± 83.2	231.5 ± 87.1	NS	↓ 4.2 ± 21.3
	Men	258.0 ± 94.4 ^a^	245.8 ± 82.0 ^a^	222.7 ± 86.9 ^b^	0.003	↓ 12.3 ± 21.8
	*p*-value **	NS	NS	NS		
VAT (cm^2^)	Total	175.5 ± 71.7 ^a^	158.0 ± 72.5 ^b^	148.8 ± 75.3 ^c^	<0.001	↓ 15.7 ± 18.5
	Women	165.6 ± 64.5 ^a^	150.6 ± 67.0 ^b^	141.3 ± 67.4 ^c^	<0.001	↓ 15.0 ± 18.5
	Men	192.1 ± 81.0 ^a^	170.3 ± 80.8 ^b^	161.4 ± 86.9 ^c^	<0.001	↓ 16.9 ± 18.7
	*p*-value **	NS	NS	NS		

BW, body weight; BMI, body mass index; WC, waist circumference; FFM, fat-free mass; FM, fat mass; SAT, subcutaneous adipose tissue; VAT, visceral adipose tissue; *, Friedman’s rank test; ** Mann–Whitney *U* test; NS, not significant; different letters indicate that the samples are significantly different at *p* < 0.05; ↓, decrease.

**Table 5:** Changes in lipid profile during the AntioxObesity program.

Lipid Profile	Group	Stage of the AntioxObesity		*p*-Value *	Changes %
		T0	T6		
Total cholesterol (mg/dL)	Total	198.4 ± 30.8	183.8 ± 30.7	<0.001	↓ 6.6 ± 12.7
	Women	201.6 ± 31.6	186.0 ± 28.7	<0.001	↓ 7.1 ± 10.9
	Men	193.0 ± 29.2	180.1 ± 34.0	0.04	↓ 5.9 ± 15.4
	*p*-value **	NS	NS		
HDL-cholesterol (mg/dL)	Total	51.1 ± 8.9	50.8 ± 9.1	NS	↓ 0.2 ± 13.9
	Women	53.3 ± 9.1	53.3 ± 9.6	NS	↑ 0.8 ± 16.6
	Men	47.3 ± 7.2	46.6 ± 6.6	NS	↓ 0.9 ± 7.9
	*p*-value **	NS	NS		
LDL-cholesterol (mg/dL)	Total	122.9 ± 32.0	109.9 ± 30.7	<0.001	↓ 8.4 ± 21.8
	Women	126.1 ± 32.5	111.3 ± 29.0	<0.001	↓ 9.8 ± 18.5
	Men	117.6 ± 31.1	107.5 ± 33.6	NS	↓ 6.0 ± 26.7
	*p*-value **	NS	NS		
Triglycerides (mg/dL)	Total	122.1 ± 42.8	115.8 ± 36.1	NS	↓ 1.2 ± 24.1
	Women	111.1 ± 35.5	107.3 ± 33.0	NS	↓ 0.8 ± 20.7
	Men	140.5 ± 48.2	130.0 ± 37.2	NS	↓ 1.9 ± 29.4
	*p*-value **	0.003	0.001		

*, Wilcoxon signed-rank test; **, Mann–Whitney *U* test; NS, not significant; ↓, decrease.

**Table 6:** Changes in plasma carotenoid levels according to gender, BMI categories, and FM reduction during the AntioxObesity program.

Variables	Category	β-Carotene (nM/L)				Lycopene (nM/L)				Lutein/Zeaxanthin (nM/L)			
		Stage		*p* *	Changes %	Stage		*p* *	Changes %	Stage		*p* *	Changes %
		T0	T6			T0	T6			T0	T6		
Total group (n = 75)		612.0 ± 275.1	650.6 ± 285.2	<0.001	↑ 9.0 ± 18.9	426.3 ± 172.1	446.5 ± 172.2	NS	↑ 8.3 ± 22.4	340.1 ± 124.2	384.4 ± 149.9	<0.001	↑ 13.9 ± 22.4
Sex	Women (n = 47)	620.4 ± 274.5	692.1 ± 350.3	<0.001	↑ 8.2 ± 14.9	410.2 ± 179.9	431.4 ± 169.0	NS	↑ 8.8 ± 20.1	338.8 ± 129.7	377.0 ± 139.5	<0.001	↑ 12.8 ± 20.3
	Men (n = 28)	598.1 ± 280.7	635.1 ± 304.1	NS	↑ 10.2 ± 24.3	453.4 ± 157.4	471.8 ± 177.6	NS	↑ 7.3 ± 27.5	342.4 ± 116.7	396.8 ± 167.8	NS	↑ 15.6 ± 25.9
	*p*-value **	NS	NS			NS	NS			NS	NS		
BMI (kg/m^2^)	<30 (n = 28)	607.3 ± 278.1	647.7 ± 302.2	0.03	↑ 7.5 ± 15.6	437.2 ± 194.2	458.4 ± 190.0	NS	↑ 8.9 ± 23.5	380.4 ± 135.3	441.0 ± 166.4	0.04	↑ 17.0 ± 22.1
	≥30 (n = 47)	614.7 ± 276.4	652.3 ± 278.3	0.004	↑ 9.8 ± 20.6	419.9 ± 159.3	439.4 ± 162.4	NS	↑ 7.8 ± 22.9	316.1 ± 111.8	350.7 ± 129.5	NS	↑ 12.0 ± 22.6
	*p*-value **	NS	NS			NS	NS			0.04	0.02		
FM reduction (kg)	<4.0 (n = 38)	630.6 ± 278.1	660.1 ± 291.7	NS	↑ 5.9 ± 18.2	434.4 ± 176.6	450.2 ± 176.6	NS	↑ 7.4 ± 23.2	350.9 ± 128.7	402.2 ± 151.5	NS	↑ 16.0 ± 23.0
	≥4.0 (n = 37)	593.4 ± 274.7	641.1 ± 282.2	0.001	↑ 12.0 ± 19.3	418.0 ± 169.3	442.7 ± 170.0	NS	↑ 9.1 ± 23.1	329.0 ± 120.2	366.1 ± 148.0	0.04	↑ 11.7 ± 21.9
	*p*-value **	NS	NS			NS	NS			NS	NS		

BMI, body mass index; FM, fat mass; *, Wilcoxon signed-rank test; **, Mann–Whitney *U* test; NS, not significant; ↑, increase.

**Table 7:** Plasma carotenoids changes due to FM reduction during the AntioxObesity program.

Carotenoids Changes (%)	FM Reduction (%)							*p*-Value *
	Q1 <3.0 (n = 19)	*p*-Value *	Q2 3.0–7.5 (n = 19)	*p*-Value *	Q3 7.6–13.4 (n = 19)	*p*-Value *	Q4 >13.4 (n = 18)	
∆ β-carotene	↑ 3.4 ± 17.2	NS	↑ 8.8 ± 19.0	NS	↑ 8.6 ± 16.5	0.02	↑ 15.0 ± 22.1	0.03
∆ Lycopen	↑ 6.7 ± 23.5	NS	↑ 9.4 ± 23.7	NS	↑ 14.3 ± 25.1	NS	↑ 2.3 ± 19.3	NS
∆ Lutein/zeaxanthin	↑ 17.0 ± 25.5	0.01	↑11.3 ± 20.6	NS	↑ 14.4 ± 24.5	0.04	↑ 10.1 ± 19.1	0.04
∆ Sum of carotenoids	↑ 9.1 ± 16.5	0.02	↑ 7.0 ± 10.3	0.01	↑ 8.6 ± 12.3	0.01	↑ 8.7 ± 14.7	0.04

FM, fat mass; *, Wilcoxon signed-rank test; NS, not significant; ↑, increase.

## Data Availability

The data presented in this study are available on request from the corresponding author.

## References

[1] Zhang X., Ha S., Lau H.C., Yu J. (2023). Excess body weight: Novel insights into its roles in obesity comorbidities. Semin. Cancer Biol..

[2] Keaver L., Webber L., Dee A., Shiely F., Marsh T., Balanda K., Perry I.J. (2013). Application of the UK foresight obesity model in Ireland: The health and economic consequences of projected obesity trends in Ireland. PLoS ONE.

[3] Calder P.C., Ahluwalia N., Brouns F., Buetler T., Clement K., Cunningham K., Esposito K., Jönsson L.S., Kolb H., Lansink M. (2011). Dietary factors and low-grade inflammation in relation to overweight and obesity. Br. J. Nutr..

[4] Kawai T., Autieri M.V., Scalia R. (2021). Adipose tissue inflammation and metabolic dysfunction in obesity. Am. J. Physiol. Cell Physiol..

[5] Thomas-Valdés S., Tostes M.D.G.V., Anunciação P.C., da Silva B.P., Sant’Ana H.M.P. (2017). Association between vitamin deficiency and metabolic disorders related to obesity. Crit. Rev. Food Sci. Nutr..

[6] Bohn T. (2019). Carotenoids and Markers of Oxidative Stress in Human Observational Studies and Intervention Trials: Implications for Chronic Diseases. Antioxidants.

[7] Dias M., Camões M., Oliveira L. (2009). Carotenoids in traditional Portuguese fruits and vegetables. Food Chem..

[8] Perry A., Rasmussen H., Johnson E.J. (2009). Xanthophyll (lutein, zeaxanthin) content in fruits, vegetables and corn and egg products. J. Food Compost. Anal..

[9] Abdel-Aal E.-S.M., Akhtar H., Zaheer K., Ali R. (2013). Dietary sources of lutein and zeaxanthin carotenoids and their role in eye health. Nutrients.

[10] Rodriguez-Concepcion M., Avalos J., Bonet M.L., Boronat A., Gomez-Gomez L., Hornero-Mendez D., Limón C., Meléndez-Martinez A.J., Olmedilla-Alonso B., Palou A. (2018). A global perspective on carotenoids: Metabolism, biotechnology, and benefits for nutrition and health. Prog. Lipid Res..

[11] Reboul E. (2013). Absorption of vitamin A and carotenoids by the enterocyte: Focus on transport proteins. Nutrients.

[12] Moran N.E., Mohn E.S., Hason N., Erdman J.W., Johnson E.J. (2018). Intrinsic and Extrinsic Factors Impacting Absorption, Metabolism, and Health Effects of Dietary Carotenoids. Adv. Nutr..

[13] Kurniawan R., Nurkolis F., Taslim N.A., Subali D., Surya R., Gunawan W.B., Alisaputra D., Mayulu N., Salindeho N., Kim B. (2023). Carotenoids Composition of Green Algae Caulerpa racemosa and Their Antidiabetic, Anti-Obesity, Antioxidant, and Anti-Inflammatory Properties. Molecules.

[14] Nurkolis F., Taslim N.A., Hardinsyah H. (2023). The importance of lutein-plant based nanoencapsulation studies—An effort to improve clinical studies on the stability and bioaccessibility of lutein for health vision. Clin. Nutr. ESPEN.

[15] Ribaya-Mercado J.D. (2002). Influence of dietary fat on beta-carotene absorption and bioconversion into vitamin A. Nutr. Rev..

[16] Britton G., Khachik F., Britton G., Pfander H., Liaaen-Jensen S. (2009). Carotenoids in Food. Carotenoids.

[17] Krinsky N.I., Johnson E.J. (2005). Carotenoid actions and their relation to health and disease. Mol. Asp. Med..

[18] Thomas S.E., Johnson E.J. (2018). Xanthophylls. Adv. Nutr..

[19] Umbreen H., Javid M., Riaz M., Zia-Ul-Haq M., Dewanjee S., Riaz M. (2021). Metabolism of Carotenoids. Carotenoids: Structure and Function in the Human Body.

[20] Bohn T., Bonet M.L., Borel P., Keijer J., Landrier J.F., Milisav I., Ribot J., Riso P., Winklhofer-Roob B., Sharoni Y. (2021). Mechanistic aspects of carotenoid health benefits—Where are we now?. Nutr. Res. Rev..

[21] Broekmans W.M., Berendschot T.T., Klopping-Ketelaars I.A., de Vries A.J., Goldbohm R.A., Tijburg L.B., Kardinaal A.F., van Poppel G. (2002). Macular pigment density in relation to serum and adipose tissue concentrations of lutein and serum concentrations of zeaxanthin. Am. J. Clin. Nutr..

[22] Chung H.-Y., Ferreira A.L.A., Epstein S., Paiva S.A.R., Castaneda-Sceppa C., Johnson E.J. (2009). Site-specific concentrations of carotenoids in adipose tissue: Relations with dietary and serum carotenoid concentrations in healthy adults. Am. J. Clin. Nutr..

[23] Mounien L., Tourniaire F., Landrier J.F. (2019). Anti-Obesity Effect of Carotenoids: Direct Impact on Adipose Tissue and Adipose Tissue-Driven Indirect Effects. Nutrients.

[24] Östh M., Öst A., Kjolhede P., Strålfors P. (2014). The concentration of β-carotene in human adipocytes, but not the whole-body adipocyte stores, is reduced in obesity. PLoS ONE.

[25] Sy C., Gleize B., Dangles O., Landrier J.F., Veyrat C.C., Borel P. (2012). Effects of physicochemical properties of carotenoids on their bioaccessibility, intestinal cell uptake, and blood and tissue concentrations. Mol. Nutr. Food Res..

[26] Jomova K., Valko M. (2013). Health protective effects of carotenoids and their interactions with other biological antioxidants. Eur. J. Med. Chem..

[27] Fiedor J., Burda K. (2014). Potential role of carotenoids as antioxidants in human health and disease. Nutrients.

[28] Bonet M.L., Canas J.A., Ribot J., Palou A. (2015). Carotenoids and their conversion products in the control of adipocyte function, adiposity and obesity. Arch. Biochem. Biophys..

[29] Eggersdorfer M., Wyss A. (2018). Carotenoids in human nutrition and health. Arch. Biochem. Biophys..

[30] Bonet M., Ribot J., Galmés S., Serra F., Palou A. (2020). Carotenoids and carotenoid conversion products in adipose tissue biology and obesity: Pre-clinical and human studies. Biochim. Biophys. Acta Mol. Cell Biol. Lipids.

[31] Böhm V., Lietz G., Olmedilla-Alonso B., Phelan D., Reboul E., Bánati D., Borel P., Corte-Real J., de Lera A.R., Desmarchelier C. (2021). From carotenoid intake to carotenoid blood and tissue concentrations—Implications for dietary intake recommendations. Nutr. Rev..

[32] Yamaguchi M. (2012). Role of carotenoid β-cryptoxanthin in bone homeostasis. J. Biomed. Sci..

[33] Yao N., Yan S., Guo Y., Wang H., Li X., Wang L., Hu W., Li B., Cui W. (2021). The association between carotenoids and subjects with overweight or obesity: A systematic review and meta-analysis. Food Funct..

[34] Landrier J.F., Marcotorchino J., Tourniaire F. (2012). Lipophilic micronutrients and adipose tissue biology. Nutrients.

[35] Hamułka J., Górnicka M., Sulich A., Frąckiewicz J. (2019). Weight loss program is associated with decrease α-tocopherol status in obese adults. Clin. Nutr..

[36] Branca F., Nikogosian H., Lobstein T., World Health Organization (2007). The Challenge of Obesity in the WHO European Region and the Strategies for Response. Summary.

[37] Ashwell M., Gibson S. (2016). Waist-to-height ratio as an indicator of ‘early health risk’: Simpler and more predictive than using a ‘matrix’ based on BMI and waist circumference. BMJ Open.

[38] FAO (2018). Dietary Assessment: A Resource Guide to Method Selection and Application in Low Resource Settings.

[39] Szponar L., Wolnicka K., Rychlik E. (2000). Atlas of Food Products and Dishes Portion Sizes.

[40] FoodData Central. https://fdc.nal.usda.gov/fdc-app.html#/.

[41] Jarosz M., Rychlik E., Stoś K., Charzewska J. (2020). Polish Dietary Reference Intakes—Revision.

[42] ISAK, International Society for Advancement of Kinanthropometry (2001). International Standards for Anthropometric Assessment.

[43] Stewart A., Marfell-Jones M.J., International Society for the Advancement of Kinanthropometry, International Society for the Advancement of Kinanthropometry (2011). International Standards for Anthropometric Assessment.

[44] World Health Organization (2022). WHO European Regional Obesity Report 2022.

[45] Friedewald W.T., Levy R.I., Fredrickson D.S. (1972). Estimation of the concentration of low-density lipoprotein cholesterol in plasma, without use of the preparative ultracentrifuge. Clin. Chem..

[46] Wu K., Schwartz S.J., Platz E.A., Clinton S.K., Erdman J.W., Ferruzzi M.G., Willett W.C., Giovannucci E.L. (2003). Variations in plasma lycopene and specific isomers over time in a cohort of U.S. men. J. Nutr..

[47] Hamułka J., Wawrzyniak A., Sulich A. (2012). The assessment of beta-carotene, lycopene and lutein intake by selected group of adults. Roczn PZH.

[48] Wawrzyniak A., Hamułka J., Friberg E., Wolk A. (2013). Dietary, anthropometric, and lifestyle correlates of serum carotenoids in postmenopausal women. Eur. J. Nutr..

[49] Myszkowska-Ryciak J., Harton A., Gajewska D., Bawa S. (2014). Lycopene, lutein and zeaxanthin intake in selected young women. Bromat Chem. Toksykol..

[50] Sulich A., Hamułka J., Nogal D. (2015). Dietary sources of lutein in adults suffering eye disease (AMD/Cataracts). Roczn PZH.

[51] Burrows T.L., Williams R., Rollo M., Wood L., Garg M.L., Jensen M., Collins C.E. (2015). Plasma carotenoid levels as biomarkers of dietary carotenoid consumption: A systematic review of the validation studies. J. Nutr. Metab..

[52] Toh D.W.K., Loh W.W., Sutanto C.N., Yao Y., Kim J.E. (2021). Skin Carotenoid Status and Plasma Carotenoids: Biomarkers of Dietary Carotenoids, Fruits and Vegetables for Middle-Aged and Older Singaporean Adults. Br. J. Nutr..

[53] Nagao A., Kotake-Nara E., Hase M. (2013). Effects of Fats and Oils on the Bioaccessibility of Carotenoids and Vitamin E in Vegetables. Biosci. Biotechnol. Biochem..

[54] Molteni C., la Motta C., Valoppi F. (2022). Improving the Bioaccessibility and Bioavailability of Carotenoids by Means of Nanostructured Delivery Systems: A Comprehensive Review. Antioxidants.

[55] Suzuki K., Ito Y., Inoue T., Hamajima N. (2011). Inverse association of serum carotenoids with prevalence of metabolic syndrome among Japanese. Clin. Nutr..

[56] Burrows T.L., Warren J.M., Colyvas K., Garg M.L., Collins C.E. (2009). Validation of overweight children’s fruit and vegetable intake using plasma carotenoids. Obesity.

[57] Chai W., Conroy S.M., Maskarinec G., Franke A.A., Pagano I.S., Cooney R.V. (2010). Associations between obesity and serum lipid-soluble micronutrients among premenopausal women. Nutr. Res..

[58] Kabat G.C., Heo M., Ochs-Balcom H.M., LeBoff M.S., Mossavar-Rahmani Y., Adams-Campbell L.L., Nassir R., Ard J., Zaslavsky O., Rohan T.E. (2016). Longitudinal association of measures of adiposity with serum antioxidant concentrations in postmenopausal women. Eur. J. Clin. Nutr..

[59] Granado-Lorencio F., Blanco-Navarro I., Pérez-Sacristán B., Hernández-Álvarez E. (2017). Biomarkers of carotenoid bioavailability. Food Res. Int..

[60] Olmedilla-Alonso B. (2023). Carotenoid Markers of Dietary Exposure and Nutritional Status. Nutrients.

[61] Bohn T., Desmarchelier C., Dragsted L.O., Nielsen C.S., Stahl W., Rühl R., Keijer J., Borel P. (2017). Host-related factors explaining interindividual variability of carotenoid bioavailability and tissue concentrations in humans. Mol. Nutr. Food Res..

[62] Desmarchelier C., Borel P. (2017). Overview of carotenoid bioavailability determinants: From dietary factors to host genetic variations: Carotenoid bioavailability determinants. Trends Food Sci. Technol..

[63] Marhuenda-Muñoz M., Domínguez-López I., Langohr K., Tresserra-Rimbau A., Martínez González M.Á., Salas-Salvadó J., Corella D., Zomeño M.D., Martínez J.A., Alonso-Gómez A.M. (2022). Circulating carotenoids are associated with favorable lipid and fatty acid profiles in an older population at high cardiovascular risk. Front. Nutr..

[64] Amengual J., Coronel J., Marques C., Aradillas-García C., Morales J.M.V., Andrade F.C.D., Erdman J.W., Teran-Garcia M. (2020). β-Carotene Oxygenase 1 Activity Modulates Circulating Cholesterol Concentrations in Mice and Humans. J. Nutr..

[65] Sugiura M., Nakamura M., Ogawa K., Ikoma Y., Matsumoto H., Ando F., Shimokata H., Yano M. (2008). Associations of serum carotenoid concentrations with the metabolic syndrome: Interaction with smoking. Br. J. Nutr..

[66] Suzuki K., Inoue T., Hashimoto S., Ochiai J., Kusuhara Y., Ito Y., Hamajima N. (2010). Association of serum carotenoids with high molecular weight adiponectin and inflammation markers among Japanese subjects. Clin. Chim. Acta.

[67] Ben Amara N., Tourniaire F., Maraninchi M., Attia N., Amiot-Carlin M.J., Raccah D., Valéro R., Landrier J.F., Darmon P. (2015). Independent positive association of plasma β-carotene concentrations with adiponectin among non-diabetic obese subjects. Eur. J. Nutr..

[68] Allore T., Lemieux S., Vohl M., Couture P., Lamarche B., Couillard C. (2019). Correlates of the difference in plasma carotenoid concentrations between men and women. Br. J. Nutr..

[69] Bovier E.R., Lewis R.D., Hammond B.R. (2013). The Relationship between Lutein and Zeaxanthin Status and Body Fat. Nutrients.

[70] Andersen L.F., Jacobs D.R., Gross M.D., Schreiner P.J., Williams O.D., Lee D.-H. (2006). Longitudinal associations between body mass index and serum carotenoids: The CARDIA study. Br. J. Nutr..

[71] Matsumoto M., Suganuma H., Ozato N., Shimizu S., Katashima M., Katsuragi Y., Mikami T., Itoh K., Nakaji S. (2021). Association between Serum Concentration of Carotenoid and Visceral Fat. Nutrients.

[72] Białkowska A., Górnicka M., Zielinska-Pukos M.A., Hallmann E., Hamulka J. (2023). Plasma Carotenoids and Polyphenols and Their Association with MetS: The Need for Nutritional Interventions. Antioxidants.

[73] Meléndez-Martínez A.J., Mandić A.I., Bantis F., Böhm V., Borge G.I.A., Brnčić M., Bysted A., Cano M.P., Dias M.G., Elgersma A. (2022). A comprehensive review on carotenoids in foods and feeds: Status quo, applications, patents, and research needs. Crit. Rev. Food Sci. Nutr..

[74] Olmedilla-Alonso B., Estévez-Santiago R., Mercadante A.Z. (2019). Dietary intake of carotenoids: Nutritional status assessment and the importance of considering free and esters forms in foods. Carotenoid Esters in Foods: Physical, Chemical and Biological Properties.

[75] Olmedilla-Alonso B., Rodríguez-Rodríguez E., Beltrán-de-Miguel B., Estévez-Santiago R. (2020). Dietary β-cryptoxanthin and αcarotene have greater apparent bioavailability than β-carotene in subjects from countries with different dietary patterns. Nutrients.

